# Busulfan/Cyclophosphamide Compared with Melphalan as a Conditioning Regimen for Autologous Transplantation of Multiple Myeloma: A Long-Term Assessment

**DOI:** 10.3390/jcm12196239

**Published:** 2023-09-27

**Authors:** Shiyuan Zhou, Yingying Zhai, Lingzhi Yan, Xiaolan Shi, Jingjing Shang, Depei Wu, Chengcheng Fu, Song Jin

**Affiliations:** 1 Jiangsu Institute of Hematology, The First Affiliated Hospital of Soochow University, Suzhou 215006, China; zhoushiyuan0827@126.com (S.Z.); yzhai2007@163.com (Y.Z.); yanlingzhi@suda.edu.cn (L.Y.); shixiaolan@suda.edu.cn (X.S.); rebeccasjj@163.com (J.S.); 2 Institute of Blood and Marrow Transplantation, Collaborative Innovation Center of Hematology, Soochow University, Suzhou 215006, China

**Keywords:** multiple myeloma, transplantation, busulfan, cyclophosphamide, melphalan, regimen

## Abstract

Background: Melphalan was poorly available in mainland China. The aim of this study is to explore the dose-adjusted busulfan/cyclophosphamide (BU/CY) as an alternative regimen in auto stem cell transplantation (ASCT) for multiple myeloma (MM). Methods: A total of 105 newly diagnosed MM patients undergoing ASCT during May 2012 and August 2017 were retrospectively analyzed. The BU/CY regimen was applied to 64 patients. Busulfan (9.6 mg/kg or 8.0 mg/kg in total) and cyclophosphamide (3.6 g/m^2^ or 3.0 g/m^2^ in total) were administered according to the creatinine clearance rate (CCR). A high-dose melphalan (HDMEL) regimen (200 mg/m^2^) was given to the other 41 patients. Results: At a median follow-up of 65 (1~119) months, estimated overall survival (OS) and progression-free survival (PFS) at 104 months in the BU/CY and HDMEL groups were 35.6% vs. 20.5% (*p* = 0.263) and 20.2% vs. 2.4% (*p* = 0.035), respectively. The median overall survival (OS) and PFS of the HDMEL and BU/CY groups were 55 vs. 70.5 months and 26 vs. 46.5 months, respectively. In multivariate analysis, the BU/CY regimen was found to be the only protective factor for PFS. No lethal toxicity was found in the BU/CY group, and treatment-related mortality (TRM) in 100 days was similar to the HDMEL group. Conclusions: MM patients may also benefit from the dose-adjusted BU/CY regimen.

## 1. Introduction

Multiple myeloma (MM) is a group of malignancies caused by abnormal clones of plasma cells. Its incidence ranks second in hematological malignancies, with an annual incidence of 6/100,000 [1]. Although many novel agents that improved the prognosis of MM emerged, autologous stem cell transplantation (ASCT) remains the irreplaceable treatment for long-term disease control for fit patients [2,3]. High-dose melphalan (HDMEL) represents the standard conditioning regimen before ASCT [4,5]. However, melphalan is currently not covered by basic medical insurance and is poorly available in mainland China. More than 24,000 newly diagnosed MMs were reported between 2012 and 2016, according to the national population-based analysis of the prevalence and incidence of MM in urban areas in China [6], but fewer than 3000 ASCT cases were registered with the Chinese Blood and Marrow Transplantation Registry Group (CBMTRG) in 2019 [7]. Considering that the data also contained non-MM patients, there might actually be a large number of MM patients who were fit for ASCT each year but were unable to receive this treatment. The contradiction between the increasing number of MM patients and expensive therapeutic drugs is becoming increasingly prominent. Therefore, it is particularly important to find a suitable protocol to be used as an alternative to HDMEL as a conditioning regimen before ASCT.

Busulfan is the most commonly used drug for hematopoietic stem cell transplantation [8]. Several studies have confirmed that the conditioning regimen containing busulfan is equivalent to HDMEL in patients with MM in ASCT [9,10,11,12]. A number of investigations have explored the role of busulfan and the cyclophosphamide (BU/CY) regimen in MM allogeneic hematopoietic stem cell transplantation (allo-HSCT) and proved to be safe and reliable [13]. Meanwhile, the application of a conditioning regimen containing busulfan and cyclophosphamide has also been reported in MM autologous transplantation [14,15]. However, there are rare studies using busulfan and cyclophosphamide as the only agents in the conditioning regimen ASCT for MM. In patients over 60 years, dose-reduced BU/CY was proved to be safe in ASCT for non-Hodgkin’s lymphoma [16]. In our previous research, we demonstrated the safety and efficacy of the BU/CY regimen in a small cohort [17,18]. In this research, we expanded the research cohort and extended the follow-up to further compare the two groups of newly diagnosed MM patients who received a dose-adjusted BU/CY and HDMEL conditioning regimen followed by ASCT.

## 2. Materials and Methods

### 2.1. Patients and Definitions

105 consecutive MM patients (56 men (53.3%) and 49 women (46.7%), median age 52 years (range 29~68), who underwent ASCT in our institution from May 2012 to August 2017 were analyzed in this retrospective study. All the patients were diagnosed according to the criteria of international myeloma working group (IMWG) [19]. Staging of MM was according to both Durie & Salmon(DS) and international staging system (ISS) [20,21]. Assessment of the treatment response was evaluated according to the international myeloma working group (IMWG) [22]. Evaluation of the organ toxicity was according to the common terminology criteria for adverse events 4.0 (CTCAE 4.0). Neutrophil engraftment was defined as an absolute neutrophil count (ANC) ≥ 0.5 × 10^9^/L for 3 consecutive days after ASCT. Platelet engraftment was defined as platelets ≥ 20 × 10^9^/L for 7 consecutive days after transfusion after ASCT. Overall survival (OS) was defined as the time interval from transplantation to death or termination of follow-up. Progression-free survival (PFS) was defined as the time interval between transplantation and disease progression or death. Patients were followed up every 3 months, and the efficacy was evaluated with reference to the Chinese guidelines for the diagnosis and treatment of multiple myeloma (revised in 2015).

### 2.2. Pre-ASCT Treatment

All the patients have received at least 2 cycles (median 4 cycles, range 2~7) of chemotherapy before transplantation. 40 (38.1%) of the patients were in complete response (CR) status at the time of transplant, 41 (39%) were in very good partial response (VGPR), 19 (18.1%) were in partial response (PR), and 5 (4.8%) had stable disease (SD) status. Triplet chemotherapies based on bortezomib and dexamethasone were given to 86 patients (81.9%) as the first line induction therapy, in which 75 (87.2%), 4 (4.7%), and 7 (8.1%) were combined with doxorubicin (or liposomal doxorubicin), cyclophosphamide, and thalidomide, respectively. Approximately 19 (18.1%) patients were given other chemotherapy.

### 2.3. Stem Cell Mobilization

Mobilizing chemotherapy consisting of CTX 3.0 g/m^2^ × 1 d was given to the patients before stem cell collection. Granulocyte colony stimulating factor (G-CSF) (10 ug/kg/d) was given for the mobilization of stem cells.

### 2.4. Transplantation Regimen and Post-ASCT Treatment

As melphalan was expensive and not covered by basic medical insurance, patients were informed to decide their conditioning regimen before ASCT according to their economic situations. Those who could not afford melphalan were enrolled in the dose-adjusted BU/CY regimen group. In the dose-adjusted BU/CY regimen, busulfan (0.6 mg/kg for those with a creatinine clearance rate [CCR] ≥ 60 mL/min and 0.5 mg/kg for those with a CCR ≥ 40 mL/min and <60 mL/min) was administered q6 h on days −7~−4 (9.6 mg/kg and 8.0 mg/kg in total, respectively) and cyclophosphamide (1.8 g/m^2^ for those CCR≥ 60 mL/min and 1.5 g/m^2^ for CCR ≥ 40 mL/min and <60 mL/min) was given once on days −3~−2. Both busulfan and cyclophosphamide were given intravenously. Sodium vedproate (1200 mg/d ivgtt. CI24 h, days −8~−4) and mesna (2.1 g/m^2^ in three divided doses, q8 h, days −4~−3) were given for seizures and hemorrhagic cystitis prophylaxis, respectively. Ursodeoxycholic acid was used for veno-occlusive disease (VOD) prophylaxis. In the HDMEL regimen, melphalan (200 mg/m^2^) was given one dose on day 2. Patients who did not reach CCR ≥ 40 mL/min at the time of transplantation were not enrolled in this study. After ASCT, all patients received maintenance therapy based on bortezomib or thalidomide. Bortezomib was given 1.3 mg/m^2^ i.h. per two weeks, and thalidomide was 100 mg p.o. qd until disease progression.

### 2.5. Statistical Analysis

All analyses were performed using SPSS v25.0. *p*-values of <0.05 were considered statistically significant. OS and PFS curves were estimated using the Kaplan-Meier method with the log-rank test. OS was defined as the time from the date of ASCT to death for any cause. PFS was defined as the time from the date of ASCT to death or disease progression. Cox regression was used for univariate and multivariate analyses of OS and PFS.

## 3. Results

### 3.1. Patient Characteristics

The baseline characteristics of the patients are shown in Table 1. There were 64 (61%) patients in the BU/CY group and 41 (39%) in the HDMEL group. No significant difference was found between these two groups in gender, M-protein, ISS stage, DS stage, pre-ASCT therapies, remission status before transplantation, hematopoietic cell transplantation-specific comorbidity index (HCT-CI), or the CD34+ cells contained in the infused grafts.

### 3.2. Engraftment

No graft failure occurred. In the BU/CY group, a median of 3.34 × 10^6^ CD34+ cells per kg (range 1.40~15.56 × 10^6^) of stem cells were infused at the time of the transplant, compared with 3.60 × 10^6^ CD34+ cells per kg (range 1.34~13.15 × 10^6^) in the HDMEL group. (*p* > 0.05) Patients achieved neutrophil engraftment at a median of 10 days (range 8~17 days) and platelet engraftment at a median of 11 days (range 8~21) in the BU/CY group, compared with 11 days (range 8~13 days) of neutrophil engraftment and 11 days (range 7~19 days) of platelet engraftment in the HDMEL group, respectively. There was no significant difference in neutrophil or platelet engraftments between the two groups (Table 2).

### 3.3. Transplant Associated Events

From the beginning of the transplant regimen to +100 days after ASCT, no significant difference was found in the presence of pneumonia, mucositis, abnormal liver function, or abnormal renal function between the BU/CY group and the HDMEL group (Table 2). No VOD was observed during the research. Fever occurred in 39 (60.9%) patients in the BU/CY group and 22 patients (53.7%) in the HDMEL group, among which 10 patients were found to have blood stream infection (BSI) in the BU/CY group, compared with 1 (2.4%) in the HDMEL group. (*p* = 0.047) There was no significant difference in neutrophil or platelet engraftments between the two groups. Treatment-related mortality (TRM) in 100 days for the two groups was similar. There was one patient who died during the period of transplantation in both groups, respectively, and both deaths were considered due to spontaneous intracranial hemorrhage.

### 3.4. Response Improvement after Transplantation

**Figure 1:**
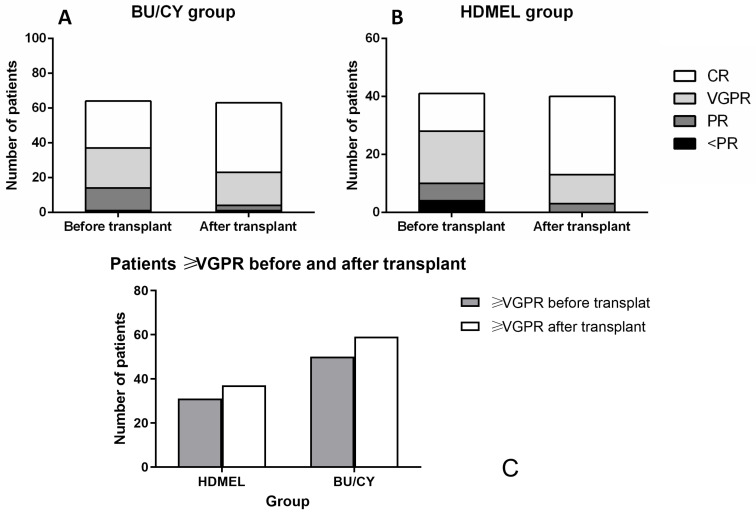
Remission status before and after transplantation in two groups. (**A**,**B**) There were 27 (42.19%) patients in CR and 22 (35.94%) in VGPR status before transplantation in the BU/CY group, compared with 13 (31.71%) in CR and 18 (43.90%) in VGPR in the HDMEL group. After transplantation, there were 40 (62.50%) patients in CR and 19 (29.69%) in VGPR status in the BU/CY group, compared with 27 (65.85%) in CR and 10 (24.39%) in VGPR in the HDMEL group. (**C**) After transplantation, there were 59 (93.7%) patients with in ≥VGPR status after transplantation in the BU/CY group and 37 (92.5%) in the HDMEL group.

The remission status before and after transplantation in the two groups is shown in Figure 1. In both groups, it was shown that more patients reached ≥VGPR status after transplantation. In the BU/CY group, there were 50 (78.1%) patients with ≥VGPR status before transplantation and 59 (93.7%) after transplantation. In the HDMEL group, 31 (75.6%) patients reached ≥ VGPR before transplantation and 37 (92.5%) after transplantation.

### 3.5. Survival of Patients in Two Groups

**Figure 2:**
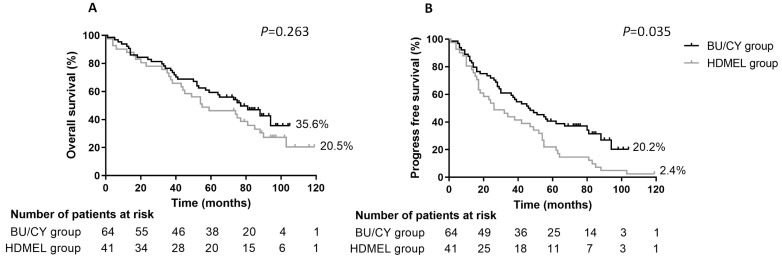
Overall survival (OS) and progression-free survival (PFS) of two groups from day 0 of the transplant. (**A**) OS in the BU/CY group showed no significant difference compared with the HDMEL group; (**B**) PFS in the BU/CY group was superior to the HDMEL group (*p* = 0.035).

**Figure 3:**
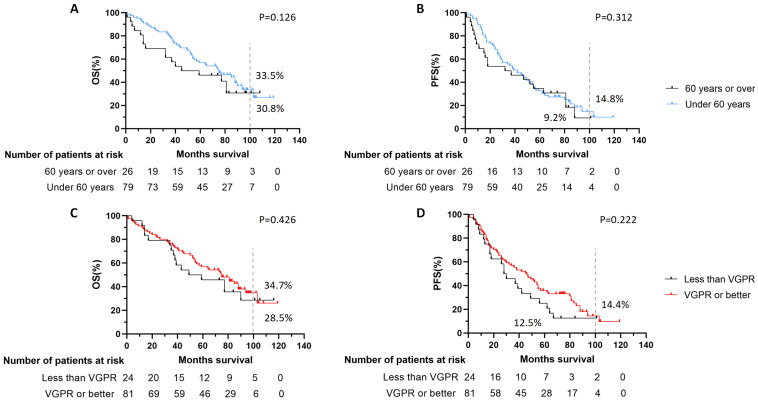
OS or PFS curves grouped by age (≥60 vs. <60) and remission status before ASCT (≥VGPR vs. <VGPR). (**A**,**B**) When the whole cohort was divided according to the age, no significant difference was found of OS or PFS between patients over or under 60 years. (**C**,**D**) When the cohort was divided according to the remission status before ASCT, no significant difference was found of OS or PFS between patients with the status better than VGPR or not.

The OS and PFS curves are shown in Figure 2. Bortezomib and thalidomide were given as maintenance therapy in all patients, and the therapy did not differ between the two groups. After a median follow-up of 65 months (range 1–119), disease progression was observed in 62 patients in the entire cohort, among which 34 were in the BU/CY group and 28 were in the HDMEL group. A total of 64 patients died, among which 34 and 30 deaths were in the BU/CY group and HDMEL group, respectively (*p* = 0.044, Table 3). The analysis of the causes of deaths showed no significant differences between the two groups. The estimated OS at 104 months of the BU/CY group and HDMEL group was 35.6% vs. 20.5%, respectively. The difference between the curves of the two groups was not statistically significant (*p* > 0.05). The estimated PFS at 104 months was 20.2% in the BU/CY group and 2.4% in the HDMEL group, respectively. The median OS and PFS of the two groups were 55 vs. 70.5 months and 26 vs. 46.5 months, respectively. A significant difference in PFS was observed in the two groups (*p* = 0.035). When the patients were grouped according to age (≥60 vs. <60) and remission status before ASCT (≥VGPR vs. <VGPR), no significant difference was found in OS or PFS curves (Figure 3).

### 3.6. Multivariate Analysis of OS and PFS

Age, gender, conditioning regimen, HCT-CI, lactate dehydrogenase (LDH) at diagnosis, disease status before ASCT, ISS staging, and DS staging were considered potential risk factors and enrolled in the analysis. In the univariate analysis of PFS, gender, conditioning regimen, and disease status before ASCT were found to be *p* < 0.2 and enrolled in the multivariate analysis. The BU/CY regimen was found to be the only protective factor in the multivariate analysis of PFS (Table 4). No variable was found to be a significant risk factor in the univariate analysis of OS for the patients; therefore, a multivariate analysis was not carried out (Table 5).

## 4. Discussion

In our previous study, we proved the safety and efficacy of the application of the BU/CY regimen and demonstrated its non-inferiority to the HDMEL regimen [17,18]. In the current study, we expanded the data sets by retrospecting more patients, extending the follow-up period, and further confirming these findings. BU/CY regimen was even found to be the only protective factor for PFS when multivariate analysis was performed. Although HDMEL is still the accepted standard regimen for MM, the results obtained from this study show that patients for whom melphalan is unavailable can still achieve efficacy no worse than HDMEL.

High-dose melphalan has been recognized as the classic conditioning regimen before ASCT for MM; however, the use of melphalan with increasing doses can cause lethal toxicity in some patients [23,24]. Thus, the regimen is innovating. Lahuerta JJ et al. pointed out that BU (12 mg/kg) combined with melphalan (MEL) (140 mg/m^2^) as a conditioning regimen for ASCT of MM can extend PFS compared to traditional HDMEL protocols [9]. Subsequently, this opinion was further supported by a prospective study conducted by Bashir et al. [25].

Myeloablative regimens containing busulfan and cyclophosphamide before allo-HSCT are widely used for acute and chronic leukemia. However, the experience with ASCT was limited. BU/CY conditioning regimen before ASCT was tried in advanced MM in 1994, and the author has indicated that this regimen was tolerable and effective in such patients [26]. In the following years, the BU/CY regimen was conducted by Amir A et al. and G Talamo et al. in their research, and similar outcomes were found [27,28]. Other scholars have added thiotepa and etoposide to the BU/CY regimen, and these studies have also obtained favorable results [14,29]. Interestingly, in our previous study [18], we did not find a superior PFS of the BU/CY regimen compared to HDMEL at a shorter follow-up; however, we did find it at a longer median follow-up of 65 months in this study, suggesting that BU/CY may have a potential advantage in PFS in long terms after ASCT.

In the use of a BU-containing conditioning regimen with a myeloablative dose, the highly virulent side effect that requires being vigilant is hepatic veno-occlusive disease (VOD). According to statistics, the incidence of VOD is about 5% [30]. In our study, no VODs occurred among 64 patients undergoing the BU/CY regimen during tranplatation. We also compared the toxic side effects, including pneumonia, fever, liver function damage, renal dysfunction, mucositis, and the time of neutrophil and platelet reconstitution during the transplant process, between the two groups, finding no significant difference. The rate of BSI in the BU/CY group has been observed to be higher than that in the HDMEL group, indicating that infection should be carefully examined during the course of the regimen. There was no significant difference in the incidence of other toxicities in the BU/CY group compared to the HDMEL group, and the hematopoietic reconstitution time was not inferior to the HDMEL group, suggesting that the BU/CY regimen did not increase lethal toxicity or prolong the hematopoietic reconstitution.

Due to the limitation of the period when the patients were transplanted, therapeutic drug monitoring (TDM) of busulfan, a more appropriate method for guiding the dose of this drug [31], was not available in our institute. Thus, the patients with impaired CCR were considered to require an empiric adjustment of the busulfan dose. Although the BU/CY regimen was considered safe without TDM monitoring in this study, we look forward to a more detailed study in the future, especially for the patients with impaired CCR. In addition, minimal residual disease (MRD) and cytogenetics data were missing due to the laboratory, which also limited the credibility of our study.

## 5. Conclusions

In summary, our study proposes that the BU/CY regimen is safe and applicable in MM. The patients may even benefit from a dose-adjusted BU/CY regimen in PFS. However, our study is limited because it is a single-center, retrospective, and non-randomized study. The missing data mentioned above is also a limitation of this study. Thus, prospective, randomized, large-sampled, and more detailed studies are warranted to validate the findings in our study.

## Figures and Tables

**Table 1:** Patient characteristics.

Characteristics	BU/CY Group (*n* = 64)	HDMEL Group (*n* = 41)	*p* Value
Age (Median, range)	52 (29–68)	52.5 (30–68)	0.583
Gender (male)	35 (54.7%)	21 (51.2%)	0.841
Myeloma subtype			
IgG	32 (50.0%)	24 (58.5%)	0.981
IgA	15 (23.4%)	9 (22.0%)	
IgM	0 (0.0%)	1 (2.4%)	
IgD	2 (3.1%)	0 (0.0%)	
Light chain	15 (23.4%)	7 (17.1%)	
Stage III at diagnosis (ISS)	16 (25.0%)	11 (26.8%)	0.743
Stage III at diagnosis (DS)	53 (82.8%)	30 (73.2%)	0.261
DS Stage B–Renal function	8 (12.5%)	7 (17.1%)	0.574
LDH at diagnosis (Abnormal) (Abnormal)	18 (28.1%)	7 (17.1%)	0.244
Therapies before ASCT (Median, range)	4 (2–7)	4 (2–8)	0.081
Time from diagnosis to ASCT (Median months, range)	7 (4–13)	7 (4–19)	0.318
Remission status before ASCT (CR)	27 (42.2%)	13 (31.7%)	0.223
HCT-CI (≥2)	4 (6.3%)	4 (9.8%)	0.421
CD34^+^ cells Transfused (Median × 10^6^/kg, range)	3.34 (1.40~15.56)	3.60 (1.34~13.15)	0.811

**Table 2:** Transplantation associated events in two groups.

Events	BU/CY Group (*n* = 64)	HDMEL Group (*n* = 41)	*p* Value
Pneumonia	12 (18.8%)	6 (14.6%)	0.791
Mucositis (Grade 2–3)	51 (79.7%)	33 (80.5%)	1.000
Abnormal liver function (Grade 2–3)	19 (29.7%)	6 (14.6%)	0.101
Abnormal renal function	22 (34.9%)	20 (48.8%)	0.220
Fever	39 (60.9%)	22 (53.7%)	0.544
BSI (blood stream infection)	10 (25.6%)	1 (2.4%)	0.047
Neutrophil engraftment (Median, range)	10 (8~17)	11 (8~13)	0.100
Platelet engraftment (Median, range)	11 (8~21)	11 (7~19)	0.147
VOD (veno-occlusive disease)	0	0	--
Deaths during this period *	1 (1.6%)	1 (2.4%)	0.751

* “This period” refers to the period from beginning of the regimen to +100 days after ASCT.

**Table 3:** Outcome event of two groups.

Events	BU/CY Group (*n* = 64)	HDMEL Group (*n* = 41)	*p* Value
Disease progression	34 (53.1%)	28 (68.3%)	0.156
Deaths	34 (53.1%)	30 (73.2%)	0.044
Cause of death			
Disease progression	24 (70.6%)	18 (60.0%)	0.435
Other causes	10 (29.4%)	12 (40.0%)	

**Table 4:** Multivariate analysis of PFS.

	Univariate Analysis			Multivariate Analysis		
Variate	RR	95%CI	*p* Value	RR	95%CI	*p* Value
Age (≥60)	1.160	0.706–1.905	0.558			
Gender (Male)	0.716	0.458–1.118	0.142	0.661	0.420–1.042	0.074
Conditioning regimen (BU/CY)	0.563	0.366–0.866	0.009	0.546	0.354–0.842	0.006
HCT-CI (≥2)	1.058	0.677–1.653	0.805			
LDH at diagnosis (Abnormal)	0.925	0.554–1.545	0.767			
Disease status before ASCT (VGPR or better)	0.705	0.429–1.160	0.169	0.641	0.386–1.063	0.085
ISS (Stage III)	1.313	0.804–2.145	0.276			
DS (Stage III)	1.384	0.816–2.348	0.229			

**Table 5:** Multivariate analysis of OS.

	Univariate Analysis			Multivariate Analysis		
Variate	RR	95%CI	*p* Value	RR	95%CI	*p* Value
Age (≥60)	1.322	0.759–2.303	0.325			
Gender (Male)	0.954	0.582–1.564	0.954			
Conditioning regimen (BU/CY)	0.732	0.447–1.200	0.216			
HCT-CI (≥2)	1.422	0.861–2.349	0.169			
LDH at diagnosis (Abnormal)	0.673	0.359–1.262	0.217			
Disease status before ASCT (VGPR or better)	0.823	0.467–1.451	0.501			
ISS (Stage III)	1.225	0.700–2.145	0.478			
DS (Stage III)	1.304	0.706–2.407	0.397			

## Data Availability

The datasets analyzed during the current study are available from the corresponding author on reasonable request.
